# Supplementary material for: Multiple Testing of Mix‐and‐Match Feature Sets in Multi‐Omics
Source: Stat Med. 2026 Jan 22;45(1-2):e70367. doi: 10.1002/sim.70367 (PMC12825407; doi:10.1002/sim.70367)
Supplement: Supplementary file 1 — Data S1: Supporting Information. [file SIM-45-0-s001.pdf]

Supplementary Material
Multiple testing of mix-and-match feature sets in multi-omics

Mitra Ebrahimpoor[1], Renée Menezes[2], Ningning Xu[1], and Jelle J. Goeman[1]

[1]Department of Biomedical Data Sciences, Leiden University Medical Center, Leiden, The Netherlands
[2]Biostatistics Centre, Department of Psychosocial Research and Epidemiology, Netherlands Cancer Institute, Amsterdam, The Netherlands

Supplementary Tables - Datasets, feature sets, run time

Supplementary Table 1 presents number of features and samples for each data used in the example analysis, and Supplementary Table 2 presents the number of feature sets in the database along with the number of feature sets available in the dataset being analyzed.

**Cancer Type**	**Samples**	**Data Type**	**Features**
BRCA	173	Gene Expression	74,985
		DNA Copy Number	30,000 loci
CRC	12	Gene Expression	75,085
		DNA Copy Number	30,000 loci

Supplementary Table 1: Details of Datasets

Data type	Database type	Feature sets in database	Feature sets used for BRCA data	Feature sets used for CRC data
Gene Expression	Hallmark pathways	50	50	50
DNA Copy Number	Chromosome arm	48	39	39
	Chromosome band	862	802	-

Supplementary Table 2: Details of Feature sets

Supplementary Table 3 presents a summary of computation time for analysis of the BRCA dataset where two-way feature sets (1950) are defined by Hallmark pathways (rows, GE) and chromosome arm (columns, CN). Runtime is distinguished by the size of feature set (GE set size and CN set size) and Algorithm type. Steps is set to 1 when both rTDP and cTDP were calculated using the Single-step shortcut (algorithm 1). Steps are set to ¿1 to represent cases where one or both of these metrics were calculated based on Algorithm 2. The time is reported in minutes. Supplementary Table 4 represents same results for CRC dataset.

GE set size	CN set size	Steps	n	Mean	SD
< 300	< 500	1	428	0.59	0.40
< 300	< 500	> 1	22	41.92	21.34
< 300	500-1000	1	404	2.65	1.63
< 300	500-1000	> 1	16	384.29	253.71
< 300	> 1000	1	244	9.54	7.50
< 300	> 1000	> 1	56	1456.03	848.40
> 300	< 500	1	277	0.62	0.38
> 300	< 500	> 1	23	41.31	24.07
> 300	500-1000	1	249	3.00	1.97
> 300	500-1000	> 1	31	406.92	272.93
> 300	> 1000	1	134	10.37	7.21
> 300	> 1000	> 1	66	1413.62	777.56

Supplementary Table 3: Computation time by algorithm and feature sets size - BRCA

GE set size	CN set size	Steps	n	Mean	SD
< 300	< 500	1	447	0.44	0.30
< 300	< 500	> 1	18	25.79	19.34
< 300	500-1000	1	424	2.02	1.48
< 300	500-1000	> 1	10	397.87	387.30
< 300	> 1000	1	291	8.38	6.04
< 300	> 1000	> 1	19	668.27	513.60
> 300	< 500	1	266	0.50	0.27
> 300	< 500	> 1	19	30.58	20.49
> 300	500-1000	1	170	8.89	5.1
> 300	500-1000	> 1	20	902.85	500.42
> 300	> 1000	1	250	2.33	1.59
> 300	> 1000	> 1	16	258.81	182.89

Supplementary Table 4: Computation time by algorithm and feature sets size - CRC

Supplementary Figures - simulation results

Details of the simulation experiments are presented in Section 6 of the main manuscript. Here, we provide figures for small and large feature sets. The general interpretation of the results is the same as for the smaller feature sets.

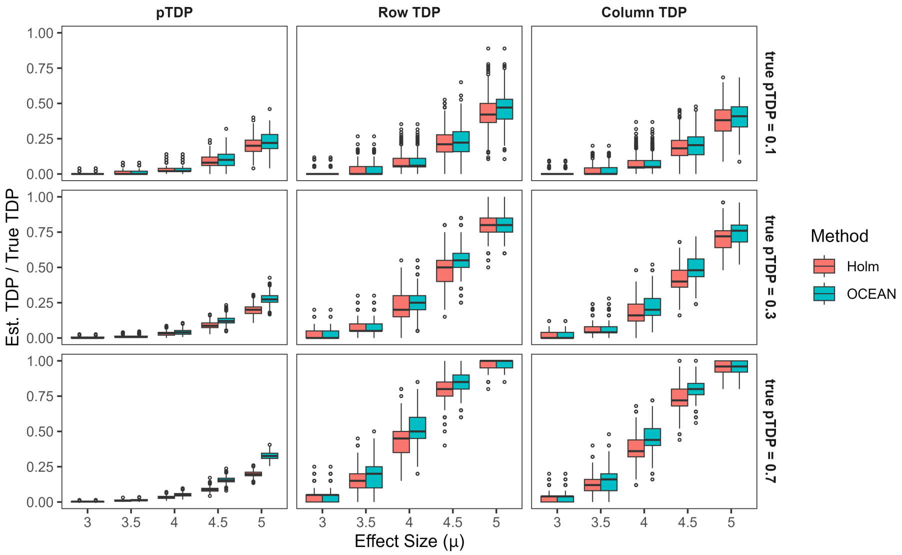

Supplementary Figure 1: Simulation experiment results (small feature set). Ratio of estimated to true TDP across effect sizes (μ) for pair, row, and column levels for a small feature set (20×25).

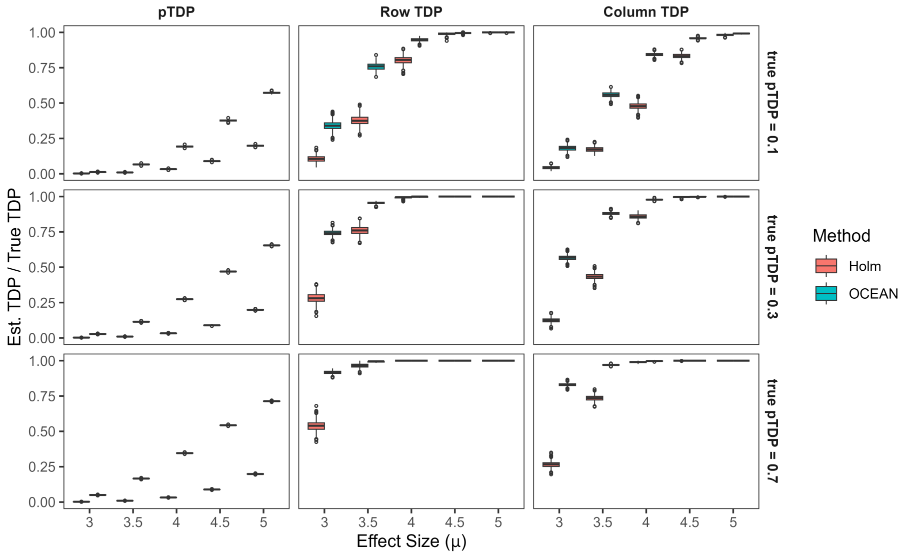

Supplementary Figure 2: Simulation experiment results (large feature set). Ratio of estimated to true TDP across effect sizes (μ) for pair, row, and column levels for a large feature set (200×500).

Supplementary Figures - Application

A detailed description of the datasets and analysis procedures is given in Section 7 of the main manuscript. Here we provide some additional figures.

Scatter plot of TDPs with similar scaling.

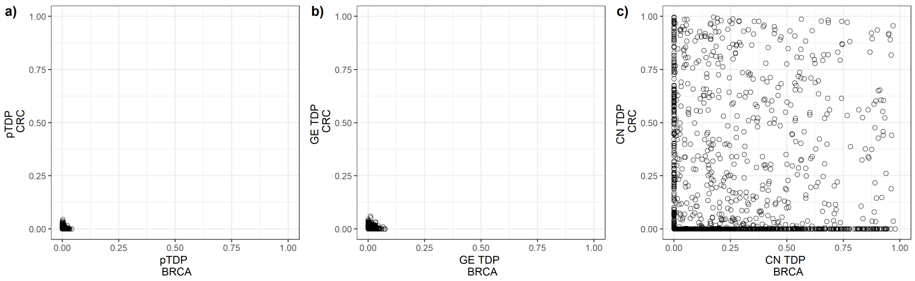

Supplementary Figure 3: Scatter plots of TDP at three levels for CRC against BRCA. a) pairwise TDP, b) GE TDP, c) CN TDP. The data are aggregated by hallmark pathways for GE and by chromosome arm for CN.

Heatmap of GE TDP aggregated by hallmark pathways (rows) and chromosome arms (columns) for CRC in terms of GE TDP (a) and CN TDP (b).

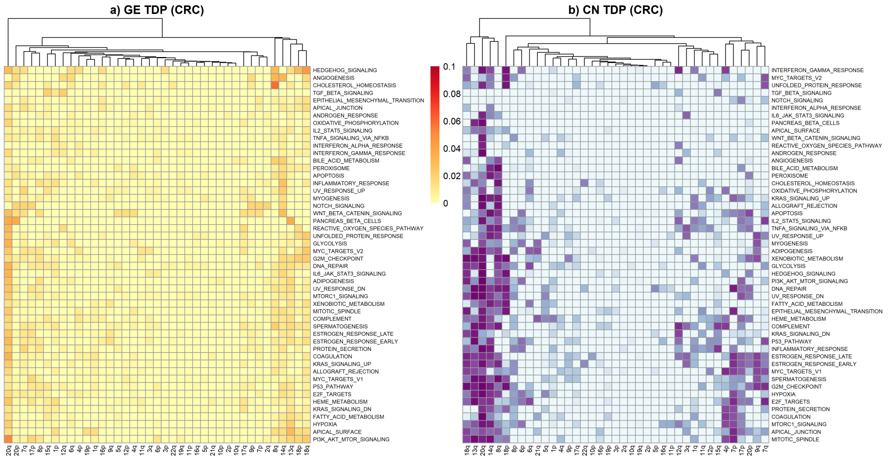

Supplementary Figure 4: Hierarchically clustered heatmap of a) GE TDP and b) CN TDP for CRC.

GE TDP and CN TDP for BRCA, with focus on q5 chromosome arm.

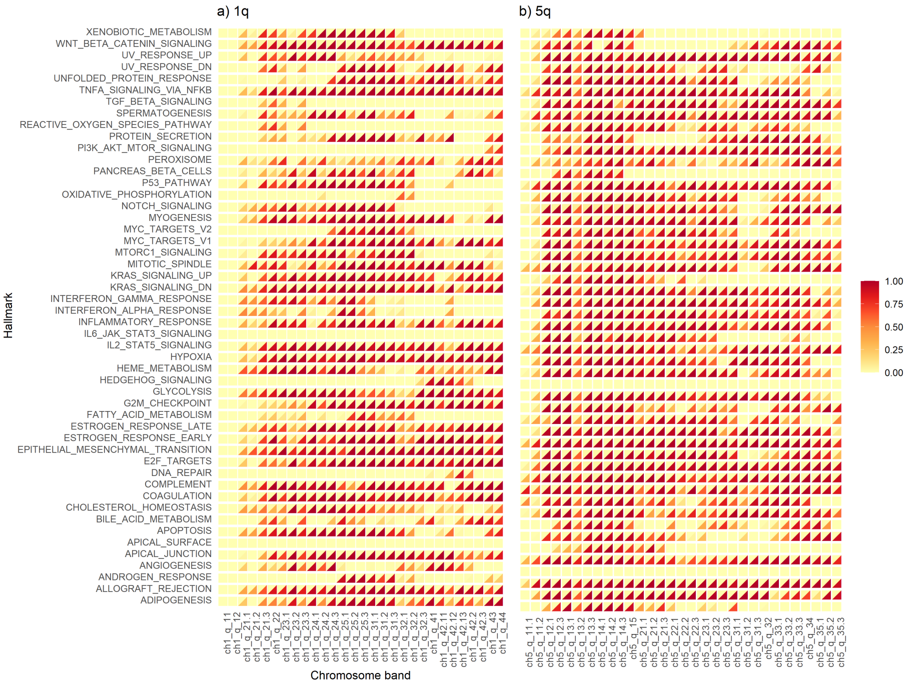

Supplementary Figure 5: Correlation matrix of BRCA results with GE TDP on the upper triangle and CN TDP on the lower triangle; CN measurements are aggregated by chromosome band and GE by hallmark pathways. The figure basically zooms into 5q chromosome arm and provides details per chromosome band.
